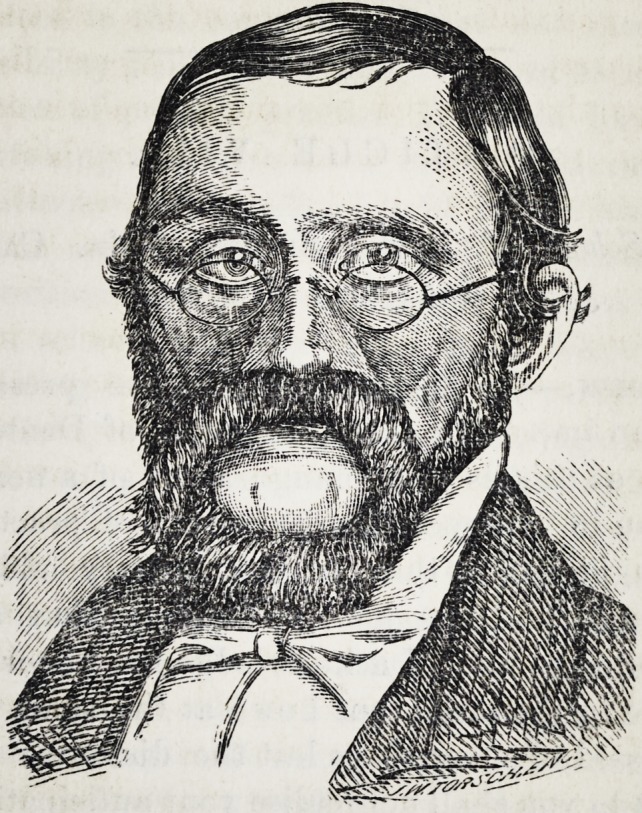# Reparation of the Face and Palate by Mechanical Means

**Published:** 1860-07

**Authors:** Wm. H. Hoopes


### ARTICLE VII.

### *Reparation of the Face and Palate by Mechanical Means.*

By WM. H. HOOPES, D. D. S.

EVERY operation, whether surgical or mechanical, which aims at a restoration of the natural appearance of the human face when a part of it has been destroyed, is attended with peculiar interest. This arises from two causes. First, deformities of the face are especially disgusting to the patient and to the beholder. Secondly, surgical appliances have to be skillfully managed, or they merely call attention to the fact that such an effort has been made without success.

The case herewith reported offered but little hope of any relief on account of the extent of the deformity; but it was undertaken and with what success may be inferred from the sketches of the face copied from photographs taken before and after the apparatus was made and adapted.

The history of the case and the present state of the affected parts are as follows:

H. R—— aged 30 years, has enjoyed good health until fifteen years ago, when he contracted primary syphilis.

About four years afterward, the disease in a tertiary form attacked the internal surface of the nasal bone and continued to spread for some five years, when fortunately its progress was arrested, though not until it had committed the most terrible destruction of the bones and soft parts of the face.

The appearance of the face presented gives but an inadequate idea of it. It may be better understood by a description. The lower margin of the nasal bones are destroyed, with the entire vomer, the nasal cartilage and a portion of the septum. The left inferior turbinated bone is gone and a portion of that of the right side. The anterior portions of the malar bones are destroyed on the left side nearly reaching the antrum. Nearly all of the superior alveolar process is gone, leaving a mere rim, with three molar teeth on one side and two on the other ; the central portion of the palatine bones is also gone, leaving an open space about the size of a half dollar piece. Of the soft parts the destruction has not been less extensive. The upper lip is destroyed, except at the angles of the mouth; ulceration had taken away much of the soft tissues of the posterior nares. The muscles of the upper lip and face that are partly destroyed are the orbicularis oris, levator labii, superioris alæque nasi, and on the left side a part of the zygomatic and levator anguli oris. It should be remembered that the sketch given reverses the side of the face.

On looking inwards and downwards, the parts presented a deep, large cavity; the motions of uvula could be seen by looking into the nose, and the tongue closed the opening through the palatine bones. Of course speech and deglutition would have been impossible, had not the patient continually kept a large piece of raw cotton in this opening. The lower lip had also begun to suffer the ravages of the fearful disease, but it was arrested at this period, and this lip presented an enlarged appearance, from the healing of a large granulated surface.

The first step in the process of making a mechanical contrivance to hide this hideous deformity, was to make a cast in plaster of the anterior portion of the face and another cast of the mouth. A gold plate was then made, fitting the roof of the mouth; upon this were inserted all the teeth that were deficient, and this plate was clasped to the remaining molar teeth. A model of an artificial nose and upper lip was then made, as near the natural form as possible. A cast of this model was filled with hard rubber, which was then vulcanized. A gold bar was attached to the inside of the artificial nose, which was made more firm by a cross bar. The opening through the palatine bones gave an opportunity to secure the nose to the plate, this was done by attaching a short tube to the plate and passing the bar through it. The plate was then placed in the mouth, the nose was attached to the face, and the bar was passed through the tube, which held it firmly in position. The stiff unnatural appearance of the upper lip was hidden by a heavy artificial moustach. The connection between the artificial and natural noses was concealed by the bow of a pair of spectacles. The artificial nose was then given a life-like color and the illusion was complete.

Baltimore, March, 1860.

## Figures and Tables

**Figure f1:**
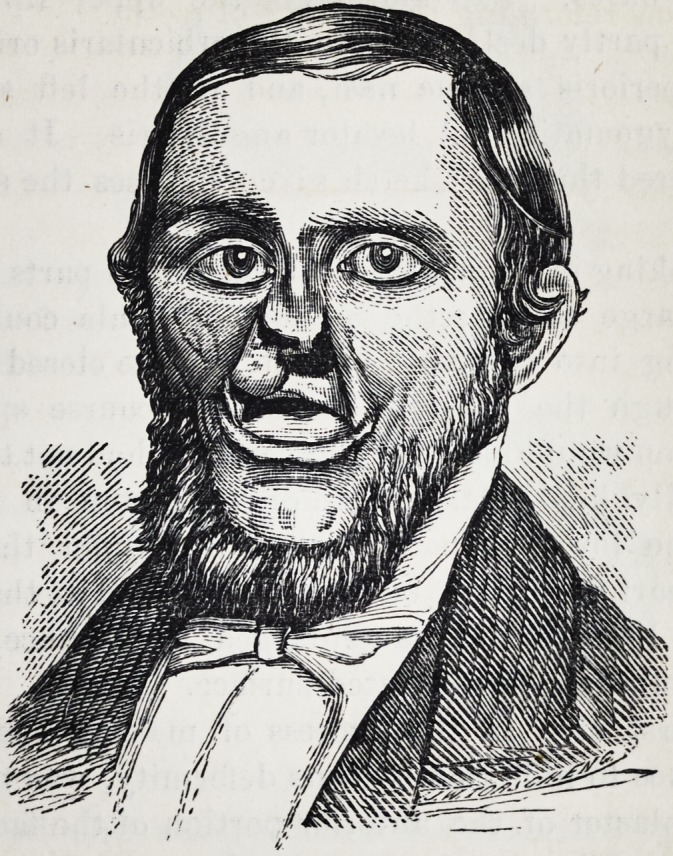


**Figure f2:**